# Conventionality matters in Chinese metaphor but not simile comprehension: evidence from event-related potentials

**DOI:** 10.3389/fpsyg.2024.1404498

**Published:** 2024-05-02

**Authors:** Yan Yu, Feng Gu, Yongqing Li, Jianghua Han

**Affiliations:** ^1^Neurocognitive Laboratory for Linguistics and Semiotics, College of Literature and Journalism, Sichuan University, Chengdu, China; ^2^Digital Convergence Laboratory of Chinese Cultural Inheritance and Global Communication, Sichuan University, Chengdu, China

**Keywords:** metaphor, simile, figurative language, cognitive processing, ERP, N400

## Abstract

Metaphor and simile, two prevalent forms of figurative language widely employed in daily communication, serve as significant research subjects in linguistics. The Career of Metaphor Theory in cognitive linguistics posits that as conventionality increases, the cognitive mechanisms of metaphor comprehension shift from “comparison” to “categorization.” In line with this notion, prior electrophysiological investigations have revealed that novel metaphors elicit a stronger N400 brain response compared to conventional metaphors. However, the observed N400 difference between conventional and novel metaphors may merely stem from the familiarity contrast between them, as conventional metaphors are typically more familiar than novel ones. To address this dichotomy, the present study not only compared the N400 responses between conventional and novel metaphors but also between conventional and novel similes. While conventional and novel similes differ in familiarity, similar to conventional and novel metaphors, both are processed via “comparison” mechanisms. The results revealed that novel metaphors elicited larger N400 amplitudes compared to conventional metaphors, aligning with previous findings. In contrast, no significant N400 differences were observed between conventional and novel similes, suggesting that familiarity disparity is unlikely to account for N400 distinctions. Our findings imply that conventional and novel metaphors undergo distinct cognitive processing mechanisms (“comparison” versus “categorization”), thereby providing further empirical validation for the Career of Metaphor Theory.

## 1 Introduction

Metaphor and simile are two of the most common types of figurative utterances. In the field of philology, metaphor and simile are considered as rhetorical devices. In cognitive linguistics, metaphor serves as a crucial cognitive tool in human thinking, which are understood via a cross-domain conceptual mapping between objects according to Lakoff and Johnson (1980). Gentner (1983) then proposed the structural-mapping theory which suggested that the mapping from source to target domain involves the relations between objects rather than the characteristics of objects. According to this theory, metaphors are understood by establishing correspondences between partially isomorphic conceptual structures of the source and target domain. The Career of Metaphor Theory (Bowdle and Gentner, 2005) further suggested that comprehending conventional and novel metaphors involve distinct cognitive processing. The understanding of novel metaphors involves comparisons, in which the source concept aligns with the target concept structurally. With using a novel metaphor repeatedly (e.g., “My job is a jail”), the conventionality will increase, then abstract metaphoric category (i.e., “the unpleasant and compulsory thing” represented by “jail”) can be created as a secondary sense of the source term (i.e., “jail”). In this case, during metaphor comprehension, the target concept (i.e., “my job”) can be aligned with the abstract metaphoric category (i.e., “the unpleasant and compulsory thing”) represented by the source term vertically. Notably, conventionality refers to the degree to which a particular metaphorical expression has become established or widely recognized within a language or culture. Conventionality is distinct from familiarity, as conventionalization encompasses not only the increasing familiarity of the expression but also the process by which a source concept acquires a metaphorical category associated with it (e.g., “jail” mentioned above referring to “the unpleasant and compulsory thing”). In short, the Career of Metaphor Theory suggested that, as conventionality increases, the understanding of metaphor will undergo a shift from comparison to categorization.

The advancement of human neuroimaging techniques, such as electroencephalogram (EEG) and functional magnetic resonance imaging (fMRI), has enabled noninvasive exploration of how the human brain processes language. This has led to the rapid growth of a new field called neurolinguistics. Similar to metaphor being a critical research focus in cognitive linguistics (Keysar et al., 2000; Gibbs et al., 2004; Garello et al., 2024), the investigation of how the human brain processes metaphor is also a significant question in neurolinguistics (Tartter et al., 2002; Iakimova et al., 2005; Desai et al., 2013; Lauro et al., 2013). Notably, Arzouan et al. (2007a) conducted an event-related potential (ERP) study to investigate brain responses to conventional metaphoric expressions (e.g., “transparent intention”) and novel metaphoric expressions (e.g., “conscience storm”). They observed that the N400 elicited by novel metaphoric expressions was enhanced compared to that elicited by conventional metaphoric expressions. The N400 amplitude difference between novel metaphor and conventional metaphor is consistently observed (Arzouan et al., 2007b; Lai et al., 2009; Goldstein et al., 2012; Tang et al., 2017a,b; Huang et al., 2022), with few exceptions (Pynte et al., 1996; Lai and Curran, 2013). Consistent observations were reported in fMRI studies, which suggested that conventionality can modulate metaphor comprehension (Cardillo et al., 2012; Horvat et al., 2022).

Arzouan et al. (2007a) suggested that the larger N400 amplitude elicited by novel metaphor expressions can be attributed to the increased difficulty of processing, as conventional metaphors are familiar while novel metaphors are unfamiliar. Tang et al. (2017a) found enhanced N400 elicited by scientific metaphors compared to conventional ones. They also proposed that the greater N400 amplitudes for scientific metaphors indicate enhanced difficulty in meaning comprehension due to their unfamiliarity. However, Lai et al. (2009) interpreted the difficulty difference between understanding novel and conventional metaphors as a result of different processing mechanisms (i.e., “comparison” in novel metaphors versus “categorization” in conventional metaphors) rather than a difference in familiarity (i.e., new utterances versus repeated utterances). Specifically, they suggested that enhanced N400 is elicited by novel metaphors because novel ways of thinking require the comparison of concepts and the creation of conceptual mappings on the spot. This view aligns with the Career of Metaphor Theory, which suggests that conventional metaphor and novel metaphor are processed by different cognitive mechanisms, i.e., “comparison” and “categorization.”

Based on the aforementioned assertions, the Career of Metaphor Theory would find support if the difference in N400 responses between novel and conventional metaphors indeed reflects distinct processing mechanisms rather than mere differences in familiarity. One strategy to explore this matter is by investigating the N400 difference between novel and conventional similes. Metaphors and similes are often discussed in conjunction, as the two types of figurative utterances share a similar typical structure: “a topic word + the linking word + a vehicle.” In typical terms, a metaphor is expressed using the copula and adheres to the structure “An X is a Y” (e.g., “The mind is a computer”). In contrast, a simile employs a comparative word such as “like” and follows the structure “An X is like a Y” (e.g., “The mind is like a computer”). It is widely accepted that understanding a simile involves a comparison mechanism because similes, like literal comparative sentences (e.g., “The daughter is like her mom”), contain explicit remarks, regardless of their level of conventionality (Bredin, 1998; Chiappe and Kennedy, 1999; Glucksberg and Haught, 2006; Lai and Curran, 2013; Gargani, 2016). Therefore, if there is no N400 difference between conventional and novel similes, it might suggest that the N400 difference between conventional and novel metaphors stems from differences in processing mechanisms rather than familiarity. Conversely, if there is an enhanced N400 response elicited by novel similes compared to conventional similes, it may indicate that the N400 difference between conventional and novel metaphors reflects differences in familiarity. The present study aims to bridge this research gap by recording ERPs in response to Chinese metaphors and similes.

In this study, the metaphor and simile expressions in Chinese were carefully selected to maintain an exact match in syntactic structure and sentence length between them. All sentences adhere to either the “X 是Y” (“是” means “is”) or “X 像Y” (“像” means “is like”) format, effectively eliminating potential confounding variables such as complex syntactic processing. Consequently, the only differing factor between metaphors and similes used in this study lies in the predicate verbs: “是” (means “is”) for metaphors and “像” (means “is like”) for similes. Therefore, Chinese metaphors and similes serve as ideal materials to investigate whether the N400 difference between novel metaphors and conventional metaphors is due to differences in their familiarity or differences in processing mechanisms, thereby contributing to the evaluation of the Career of Metaphor Theory from a neurophysiological perspective, offering insights into the underlying neurocognitive mechanisms involved in processing metaphor and simile.

## 2 Materials and methods

### 2.1 Participants

Twenty-three students from Sichuan University, who were native Mandarin Chinese speakers, participated in the study. The participant group comprised 11 men and 12 women, with an average age of 22.3 years (SD = 2.16, range: 19–27) and an average year of education of 15.9 years (SD = 1.70, range: 13–18). All participants had either normal or corrected-to-normal vision. They were free from any neurological disorders or significant head injuries and right-handed according to the Edinburgh Inventory (Oldfield, 1971). Before the experiment, participants willingly gave their consent by signing consent forms, and after the experiment, compensation was provided. The study received approval from the Biomedical Research Ethics Committee of Sichuan University. The data of five additional subjects were not included due to excessive amounts of artifacts observed during EEG recording.

### 2.2 Stimuli

The sentence stimuli used in this study were categorized into four conditions (refer to Table 1): a conventional metaphor condition (e.g., 历史是长河 - History is a long river), a novel metaphor condition (e.g., 工作是合唱 – Work is a chorus), a conventional simile condition (e.g., 历史像长河 – History is like a long river), and a novel simile condition (e.g., 工作像合唱 – Work is like a chorus). Conventional figurative sentences (conventional metaphors or conventional similes) were characterized by a higher level of familiarity, interpretability, and cloze probability of the vehicle word due to the repeated use. Novel figurative sentences (novel metaphors or novel similes) were newly constructed and characterized by a lower level of familiarity, interpretability, and cloze probability of the vehicle word. Each sentence stimulus comprised three words: a topic word, a linking word (“是” or “像,” means “is” or “is like”), and a vehicle. The topic words consisted of 2–3 Chinese characters, and all vehicles consisted of 2 Chinese characters. There were 50 sentences in each condition. The 50 conventional metaphors and 50 conventional similes shared the same topic and vehicle words, with “是” (“is”) used for metaphor sentences and “像” (“is like”) for simile sentences. This correspondence also held true for the 50 novel metaphors and 50 novel similes. Additionally, there were 250 literal sentences (e.g., 长江是长河 - The Yangtze River is a long river) and 250 anomalous sentences (e.g., 律师是长河 - The lawyer is a long river) used as filler sentences.

**Table 1:** Example materials and mean cloze probability, familiarity, and interpretability for each condition.

Type	Conventionality	Sentences	English meaning	Cloze Probability		Familiarity		Interpretability	
				Mean	SD	Mean	SD	Mean	SD
Metaphor	Conventional	历史是长河。	History is a long river.	0.01	0.04	3.96	0.50	4.39	0.36
	Novel	工作是合唱。	Work is a chorus.	0.00	0.00	1.74	0.37	2.99	0.66
Simile	Conventional	历史像长河。	History is like a long river.	0.03	0.09	4.01	0.54	4.46	0.37
	Novel	工作像合唱。	Work is like a chorus.	0.00	0.00	1.82	0.41	3.34	0.62

The 50 sentences for each condition were chosen from four larger pools, each containing 150 sentences. The authors constructed these four pools of sentences with reference to a dataset by Wang (2022). Prior to the experiment, the cloze probability of the vehicle words in the 150 sentences of each pool was assessed. Forty native Chinese speakers (mean age = 21.8 years, SD = 1.29; mean year of education = 15.8 years, SD = 1.22), college students at Sichuan University, participated in this pre-test. Sentences lacking sentence-final vehicle words were presented, and participants were instructed to write down the word that first came to mind, completing the sentence plausibly. Another group of 40 college students (mean age = 20.8 years, SD = 1.73; mean year of education = 14.8 years, SD = 1.45) was enlisted to evaluate the familiarity and interpretability of the 150 sentences in each pool, using a five-point scale (1 = highly non-familiar/non-interpretable, 5 = highly familiar/interpretable). Fifty sentences were chosen for both conventional metaphors and conventional similes because they received ratings exceeding 3 points in both familiarity and interpretability tests. Conversely, 50 sentences for both novel metaphors and novel similes were chosen because they received ratings below 3 points in the familiarity test. Table 1 presents the mean values of familiarity and interpretability for the selected 50 sentences in each condition, along with the cloze probability of the vehicle words. The familiarity values, interpretability values, and cloze probability values were analyzed by ANOVA using conventionality (conventional and novel) and figurative type (metaphor and simile) as within-subject factors. Results were summarized in Table 2, indicating significant main effect of conventionality in all the three ANOVAs. Importantly, for the familiarity values, planned paired samples *t*-tests revealed significant difference between conventional and novel metaphors [*t*(49) = 25.901, *p* < 0.001, two-tailed] and between conventional and novel similes [*t*(49) = 28.462, *p* < 0.001, two-tailed].

**Table 2:** ANOVA results of the pretest evaluations of the materials.

Measures	Factors	F	p	η2
Familiarity values	Conventionality	825.418	<0.001	0.944
	Figurative type	4.389	0.041	0.082
	Conventionality × Figurative type	0.449	0.506	0.009
Interpretability values	Conventionality	163.474	<0.001	0.769
	Figurative type	37.931	<0.001	0.436
	Conventionality × Figurative type	21.499	<0.001	0.305
Cloze probability values	Conventionality	8.237	0.006	0.144
	Figurative type	2.685	0.108	0.052
	Conventionality × Figurative type	2.685	0.108	0.052

### 2.3 Procedure

The 700 sentence stimuli, comprising 50 for each condition and 500 filler sentences (including 250 anomalous sentences and 250 literal sentences), were segmented into four blocks, each containing 175 sentences, avoiding the repetition of vehicles in each block. Within each block, there were 25 metaphors, 25 similes, and 125 fillers. The substantial number of filler sentences served two purposes: firstly, to prevent participants from recognizing that all figurative sentences were semantically congruent, thus deterring them from relying solely on sentence structure recognition as a strategy to assess interpretability. Secondly, the substantial number of filler sentences helped reduce potential repetitive effects on the processing of figurative sentences. These fillers comprised 75 non-figurative sentences using the same linking word (“是” or “像”) as figurative ones (e.g., 长江是长河 – The Yangtze River is a long river), along with 50 sentences using a different linking word (i.e., “有”) compared to figurative ones (e.g., 妹妹有玩偶 – The young sister has a toy). Each block contained a total of 112 (or 113) semantically congruent sentences and 63 (or 62) semantically incongruent sentences. The sequence of the four blocks was balanced among participants through Latin Square design. Each block had a duration of 13–15 min, and intervals of rest were provided between blocks to maintain participant comfort.

**Figure 1:**
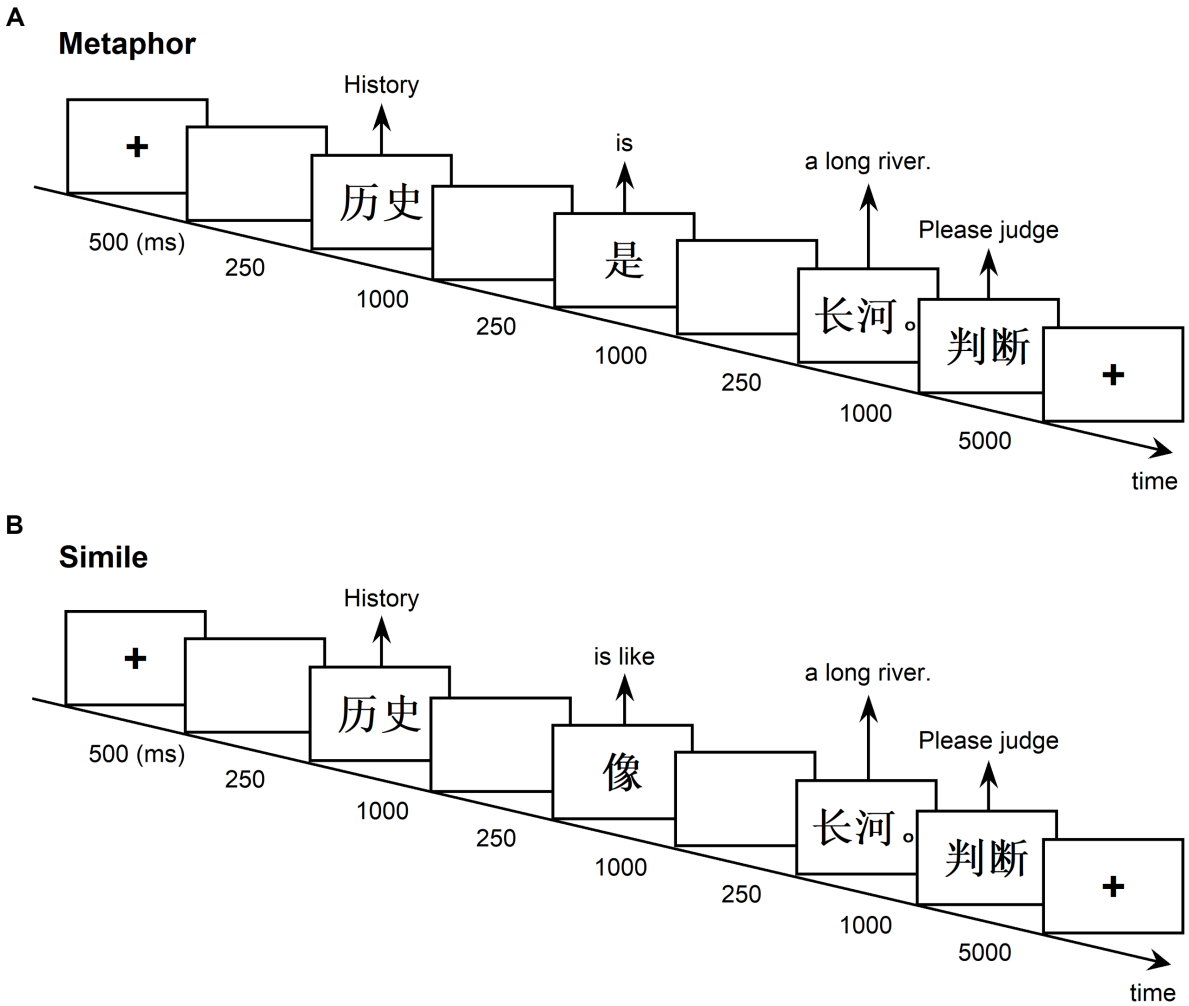
Experimental paradigm. Metaphor **(A)** and simile **(B)** sentences were presented word by word. The specified durations for each word and intervals between words were given. The end of the sentence was marked by a concluding period. Upon the emergence of the response instruction, participants were prompted to swiftly assess the interpretability of each sentence by pressing a designated key (perfect sense, some sense, little sense, and no sense). Following each participant response, the program transitioned to the next sentence.

In each block, a sentence stimulus was presented using the paradigm adapted from Tang et al. (2017a) (Figure 1). The sequence unfolded as follows: a fixation cross (500 ms), a blank screen (250 ms), the topic word (1,000 ms), another blank screen (250 ms), the linking word (“是” or “像”) (1,000 ms), followed by another blank screen (250 ms), the vehicle word (1,000 ms), and ended with the response instruction (5,000 ms). The vehicle word was accompanied by a concluding period, signifying the completion of the sentence. Participants were directed to rapidly assess the interpretability of each sentence by pressing one of four keys (i.e., “perfect sense,” “some sense” “little sense,” and “no sense”) upon the appearance of the response instruction. Following each response, the program seamlessly transitioned to the subsequent sentence. The stimuli were displayed in black against a white background, and the experiment was conducted in a quiet room with subdued lighting. Prior to the formal experiment, participants underwent a brief practice session.

### 2.4 Electroencephalograms recording

Electroencephalograms (EEG) data were acquired through a 64 Ag/AgCl electrode cap connected to a SynAmps 2 amplifier (NeuroScan, Charlotte, NC, USA). Electrode placement adhered to the international 10/20 system, with supplementary electrodes positioned at the left and right mastoids. Vertical electrooculograms (EOGs) were captured using a pair of bipolar channels situated above and below the left eye. AFz served as the grounding point, and the impedance between the reference electrode and any other electrode was maintained below 10 kΩ. Continuous EEG data were recorded and digitized at a 24-bit resolution, with a sampling rate of 500 Hz.

### 2.5 Data analysis

The offline processing of continuous EEG data recorded utilized Neuroscan (ver 4.3). Initially, the EEG data underwent filtering via a finite pulse response filter, with a bandpass range of 0.1–25 Hz. Correction for eye-blink artifacts followed a regression-based procedure outlined by Semlitsch et al. (1986). Subsequently, the EEG data were segmented into epochs, time-locked to the critical word (the vehicle word in each condition). Each epoch lasted 900 ms and included the 100 ms period preceding the vehicle word. All epochs underwent baseline correction utilizing the pre-stimulus 100-ms baseline. Channels displaying amplitudes exceeding ±75 μV were flagged for artifacts. EEG epochs with artifacts in any channel, excluding the VEOG channel, were excluded. Following artifact removal, the remaining EEG epochs were categorized based on four experimental conditions and averaged independently, resulting in the generation of ERPs for each condition. Finally, the ERPs were referenced to an average reference. SPSS (version 22) was employed to analyze both the ERP amplitude data and behavioral data.

## 3 Results

### 3.1 Behavioral results

The response times for the four experimental conditions are presented in Table 3 and were subjected to ANOVA with conventionality (conventional and novel) and figurative type (metaphor and simile) as within-subject factors. The results unveiled significant main effects of conventionality [*F*(1, 22) = 221.671, *p* < 0.0001, η^2^ = 0.496] and figurative type [*F*(1, 22) = 10.509, *p* = 0.004, η^2^ = 0.323]. Additionally, a significant interaction between the two factors was observed [*F*(1, 22) = 6.454, *p* = 0.019, η^2^ = 0.227]. *Post hoc* paired samples *t*-tests were conducted to delve into the interaction effect. These analyses disclosed a significant difference between conventional metaphor and conventional simile conditions [*t*(22) = 3.247, *p* = 0.004, two-tailed]. However, no significant difference emerged between novel metaphor and novel simile conditions [*t*(22) = 0.708, *p* = 0.487, two-tailed], contributing to the observed interaction between conventionality and figurative type.

**Table 3:** Mean response times and sensicality values for each condition.

Figurative type	Conventionality	Response time (ms)		Sensicality value	
		Mean	SD	Mean	SD
Metaphor	Conventional	654.12	288.33	3.34	0.47
	Novel	835.34	385.41	2.50	0.74
Simile	Conventional	531.09	203.63	3.64	0.41
	Novel	821.56	366.62	2.70	0.65

The sensicality values (perfect sense = 4, some sense = 3, little sense = 2, and no sense = 1) for the four experimental conditions, as presented in Table 2, underwent the same ANOVA for response time analysis. The results revealed significant main effects of conventionality [*F*(1, 22) = 106.210, *p* < 0.0001, η^2^ = 0.828] and figurative type [*F*(1, 22) = 21.510, *p* = 0.0001, η^2^ = 0.494]. There was no significant interaction between the two factors [*F*(1, 22) = 1.641, *p* = 0.213, η^2^ = 0.069].

### 3.2 ERP results

**Figure 2:**
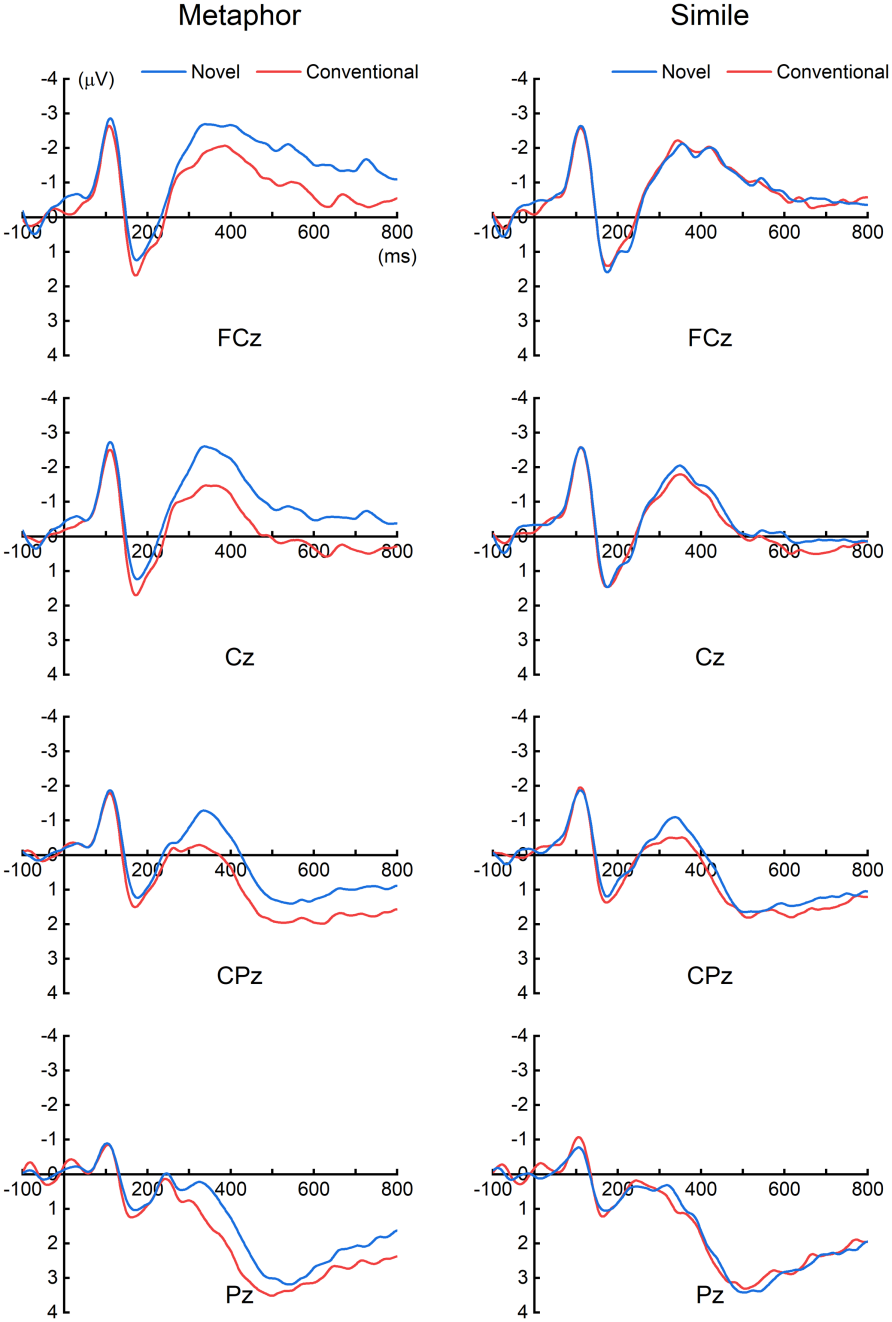
Grand-averaged ERPs elicited by the vehicle words of each condition. For the metaphor sentences, prominent ERP differences around 400 ms (i.e., N400) were observed between the conventional and novel conditions. In contrast, for the simile sentences, no prominent ERP difference was noted between the two conditions.

**Figure 3:**
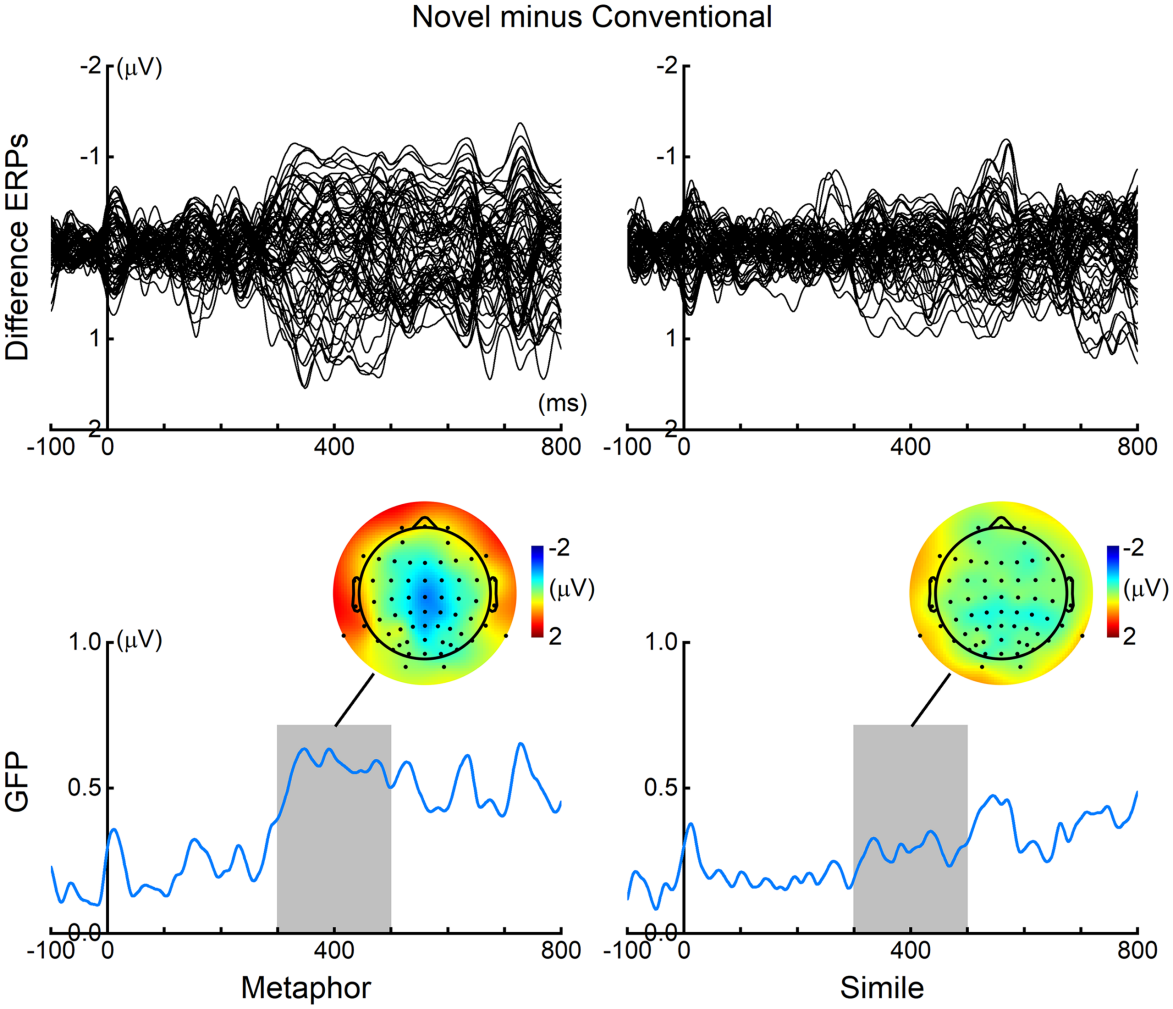
ERP differences between novel and conventional conditions. In analyzing both metaphor and simile sentences, the ERPs for the conventional condition were subtracted from those for the novel condition. The resulting difference ERPs from the 64 recording electrodes are visualized in the upper panels as butterfly plots. Concurrently, the global field power (GFP) of the difference ERPs for each condition is calculated and presented in the lower panels. For metaphor sentences, a prominent N400 response was evoked by the novel condition in comparison to the conventional condition. In contrast, there was no noticeable N400 elicited by the novel condition when compared to the conventional condition for simile sentences. The time interval selected for statistical analysis of the N400 (300–500 ms) is highlighted in gray bars. Additionally, topographic maps of the N400, calculated based on the mean ERP amplitudes within the 300–500 ms window, are visually depicted.

Figure 2 presents the grand-averaged ERPs elicited by the vehicle words in the four experimental conditions at four representative electrodes (FCz, Cz, CPz, and Pz). Notably, for the metaphor sentences, pronounced differences were observed between the conventional and novel conditions around 400 ms. In contrast, for the simile sentences, no prominent difference was noted between the two conditions. To better elucidate the ERP differences between the conventional and novel conditions, we computed the difference ERPs by subtracting the ERPs elicited in the conventional condition from those in the novel condition for both metaphor and simile sentences. The upper panels of Figure 3 depict the resulting grand-averaged difference ERPs from all 64 recording electrodes (excluding the VEOG channel). Additionally, the global field power (GFP) of each difference ERP was calculated, as illustrated in the lower panels of Figure 3. The topographic maps at the peaks of the GFP elucidate the spatial distribution of the ERP differences around 400 ms (i.e., the N400) between the conventional and novel conditions.

The current study observed a prominent N400 response in the novel metaphor condition when compared to the conventional metaphor condition (Figures 2, 3). However, the N400 response was not evident in the novel simile condition in contrast to the conventional simile condition (Figures 2, 3). For the statistical analysis of the mean N400 amplitudes, mean ERP amplitudes were computed for each condition and participant within the 300 – 500 ms time window across four electrodes (FCz, Cz, CPz, Pz). The selection of this time window and these electrodes was guided by prior knowledge indicating that N400 is prominent around 400 ms in the central area of the scalp (e.g., De Grauwe et al., 2010; Goldstein et al., 2012).

**Figure 4:**
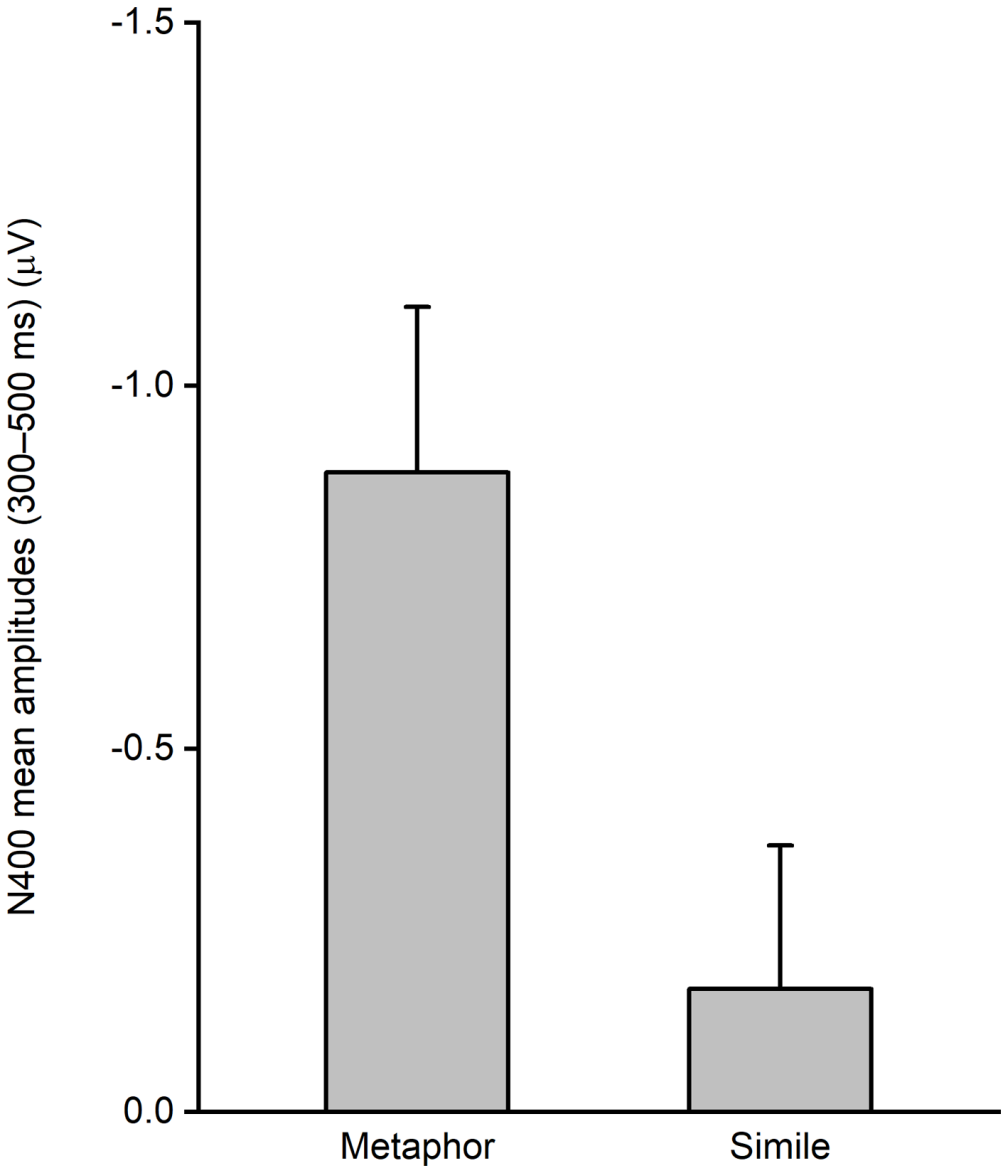
Mean N400 amplitudes. Significant N400 response was elicited by the novel condition when compared to the conventional condition for the metaphor, but no significant N400 response was elicited by the novel condition when compared to the conventional condition for the simile. Moreover, the mean N400 amplitudes were significantly larger for the metaphor compared to the simile.

For each participant, the mean N400 amplitude was derived by subtracting the mean ERPs (within 300–500 ms across electrodes FCz, Cz, CPz, and Pz) for the conventional condition from those for the novel condition, for both metaphor and simile. Paired samples *t*-test revealed a significant N400 response elicited by the novel condition when compared to the conventional condition for the metaphor sentences [*t*(22) = 3.864, *p* = 0.001, two-tailed] (Figure 4). In contrast, there was no significant N400 response elicited by the novel condition in comparison to the conventional condition for the simile sentences [*t*(22) = 0.860, *p* = 0.399, two-tailed]. Moreover, paired samples *t*-test revealed that the mean N400 amplitudes were significantly larger for the metaphor compared to the simile [*t*(22) = 2.167, *p* = 0.041, two-tailed].

## 4 Discussion

The principal finding of this study was that conventional and novel metaphors elicited distinct ERP patterns around 400 ms, whereas no significant ERP difference was observed between conventional and novel similes. These results were discussed as evidence supporting the Career of Metaphor Theory and shed light on the neurocognitive mechanisms involved in comprehending figurative utterances.

### 4.1 The N400 difference between conventional and novel metaphors

The current study found that novel metaphors elicited more negative N400 responses compared to conventional metaphors (Figures 2–4). These results were consistent with previous findings (Arzouan et al., 2007a; Lai et al., 2009; Tang et al., 2017a; Huang et al., 2022). As mentioned, the N400 difference between conventional and novel metaphors was attributed to either different familiarity or different cognitive mechanisms between the two types. In this study, similes were introduced as an additional control condition to investigate whether familiarity could modulate N400 amplitude. The results showed no significant N400 difference between conventional and novel similes. Therefore, familiarity may not be the critical factor modulating the N400 amplitude of figurative sentences. Hence, the N400 difference between conventional and novel metaphors observed in the present study and previous studies is best explained as the result of different neurocognitive mechanisms involved in conventional and novel metaphors, rather than differences in familiarity.

The N400 is widely recognized as an index linked to meaning processing (for a review see Kutas and Federmeier, 2011). Some studies suggest that the N400 reflects aspects of semantic integration processes (Kutas and Hillyard, 1980; Hagoort et al., 2004; Lau et al., 2008; Bermudez-Margaretto et al., 2015). According to this perspective, the N400 effect reflects the process of integrating critical words with the previous context. Recent new perspectives consider the N400 as a component that reflects the retrieval of lexical semantic information from long-term memory (Brouwer et al., 2012; Delogu et al., 2019; Aurnhammer et al., 2023). From this viewpoint, the N400 effect results from the enhanced activation of characteristics from long-term memory representations linked to a lexical item. Based on these perspectives, the current results suggest that semantic integration or lexical retrieval of the vehicles in the metaphors might be more challenging in the comprehension of novel metaphors (which involve comparison mechanisms) compared to conventional metaphors (which involve categorization mechanisms).

Notably, the N400 responses in the present study exhibited a longer duration (lasting from 300 ms to 800 ms, see Figure 3), compared to the typical N400 responses, which generally manifest within the 300–500 ms range. Similar prolonged durations of N400 were reported in studies of Arzouan et al. (2007a), Goldstein et al. (2012), and Tang et al. (2017a). This late negativity has been interpreted as a secondary integration of meaning, which supported the serial processing model of novel metaphor (Bowdle and Gentner, 2005; Tang et al., 2017a). As proposed by the Career of Metaphor theory, the comprehension of figurative language may involve either direct or indirect processing. The serial processing is influenced not only by conventionality but also by grammatical form. Typically, comprehension of conventional figurative sentences is direct: Conventional metaphors are processed as direct categorizations, while conventional similes are comprehended as direct comparisons. Conversely, the comprehension of novel figurative sentences can be either direct or indirect depending on grammatical structure: Novel similes are understood as direct comparisons, while novel metaphors are processed as indirect comparisons (Bowdle and Gentner, 2005). Therefore, the late negativity elicited by novel metaphors might represent a continuation of the N400, indicating the persistent difficulty in processing novel metaphors through the indirect comparison mechanism.

In summary, our findings provide additional neurophysiological evidence supporting the Career of Metaphor Theory, which suggested different cognitive mechanisms between novel and conventional metaphors (i.e., “comparison” versus “categorization”). The Career of Metaphor Theory provides a convincing theoretical framework for examining metaphors and has thus emerged as an influential theory in cognitive linguistics (Bowdle and Gentner, 2005; Jones and Estes, 2006; Thibodeau and Durgin, 2008; Jamrozik et al., 2016). It reconciles apparent contradictions between traditional comparison views and subsequently-emerged categorization views of metaphor comprehension, elucidating processing differences for metaphors of varying conventionality. Furthermore, it addresses the dichotomy between the direct access model and the serial processing claim of metaphor, proposing that processing metaphors in direct or indirect approach depends on both their levels of conventionality and their grammatical form (Bowdle and Gentner, 2005). Given its significance in cognitive linguistics, it is essential to subject the Career of Metaphor Theory to empirical scrutiny from a neurophysiological perspective using electrophysiological technology. Our findings offer such empirical support for the Career of Metaphor Theory, contributing to its evaluation from a neurophysiological standpoint.

### 4.2 No N400 difference between conventional and novel similes

The conventional and novel similes utilized in the current study exhibited significant differences in familiarity, as evidenced by Table 1, which notably influenced participants’ response times and sensicality values (Table 3). Similarly, conventional and novel metaphors also displayed such differences. However, the current study observed no significant N400 difference between conventional and novel similes, as illustrated in Figures 2–4. These results indicate that the N400 response is not significantly influenced by the familiarity disparity between conventional and novel similes. Moreover, the absence of a significant N400 difference between conventional and novel similes aligns with the perspective that both conventional and novel similes are processed by the same “comparison” mechanisms. In summary, the absence of an N400 difference between conventional and novel similes contributes to elucidate the N400 difference between conventional and novel metaphors.

Previous N400 studies have indicated that N400 amplitude is notably modulated by cloze probability (Groppe et al., 2010; Arbel et al., 2011; Zhu et al., 2019). In the current study, the difference in cloze probability between conventional and novel similes was relatively minor (0.03 versus 0.00, see Table 1) compared to previous studies. Therefore, such a slight difference in cloze probability may not be sufficient to induce an N400 difference between conventional and novel similes.

### 4.3 Different neurocognitive mechanisms between metaphor and simile

Metaphor and simile, both being figurative utterances, share a similar typical structure: “a topic word + the linking word + a vehicle.” Particularly in Chinese, these two figurative utterances exhibit close resemblance, with the only distinction lying in the linking word of metaphor and simile (“是” versus “像”). However, previous studies in cognitive linguistics have suggested that metaphor and simile are processed differently (Bredin, 1998; Bowdle and Gentner, 2005; Glucksberg and Haught, 2006; Lai and Curran, 2013). For instance, according to the Career of Metaphor Theory, novel metaphors are processed as comparisons, because novel metaphors involve source terms that only refer to a domain-specific concept but not to a domain-general concept. Thus, comprehension of novel metaphors entails comparing the source and target domains, aligning the target concept structurally with the source concept to access the metaphorical meaning. In contrast, conventional metaphors are processed through categorization, with source terms having both literal and metaphorical meanings. Consequently, comprehension of conventional metaphors involves vertically aligning the target concept with the source concept without domain comparison. On the other hand, it is generally agreed that understanding similes only requires comparison mechanisms, and there are no cognitive process differences between novel and conventional similes (Bredin, 1998; Chiappe and Kennedy, 1999; Glucksberg and Haught, 2006; Lai and Curran, 2013; Gargani, 2016).

The results of the present study align well with the perspectives proposed in cognitive linguistics as discussed above, which suggested that metaphor and simile are processed by distinct cognitive mechanisms. Specifically, the N400 difference observed between conventional and novel metaphors reflects the involvement of comparison mechanisms in the comprehension of novel metaphors, whereas categorization mechanisms are engaged in comprehending conventional metaphors. In contrast, the absence of an N400 difference between conventional and novel similes reflects the involvement of the same mechanisms (i.e., comparison mechanisms) in understanding both conventional and novel similes. Furthermore, employing the high temporal resolution ERP technique, this study elucidated that the differences in neurocognitive processing between “comparison” (simile and novel metaphor) and “categorization” (conventional metaphor) occur within the 300–800 ms timeframe.

The distinction in neurocognitive mechanisms between metaphor and simile is also supported by a previous fMRI study conducted by Shibata et al. (2012). This study observed higher activation levels in the medial frontal region for similes and more right-sided prefrontal activation for metaphors, while both conditions exhibited similar activation patterns in the left frontal region. However, this fMRI study directly compared metaphor and simile without considering the level of conventionality. The present findings, along with previous perspectives in cognitive linguistics, suggest that the level of conventionality is a critical factor that modulates the processing mechanisms underlying novel and conventional metaphors. Therefore, future investigations are needed to elucidate the neural substrates involved in the comprehension of metaphor and simile at different levels of conventionality.

## 5 Conclusion

The current study employed novel metaphors, conventional metaphors, novel similes, and conventional similes as experimental conditions to assess the Career of Metaphor Theory from a neurophysiological perspective using ERP technology. We observed a significant N400 difference between conventional and novel metaphors, while no significant N400 difference was observed between conventional and novel similes. Our findings, which differentiate between novel and conventional metaphors and assimilate novel and conventional similes, lend support to the Career of Metaphor Theory and the comparison view of simile.

## Data availability statement

The original contributions presented in the study are included in the article/Supplementary material, further inquiries can be directed to the corresponding author.

## Ethics statement

The studies involving humans were approved by the Biomedical Research Ethics Committee of Sichuan University. The studies were conducted in accordance with the local legislation and institutional requirements. The participants provided their written informed consent to participate in this study.

## Author contributions

YY: Conceptualization, Data curation, Formal analysis, Investigation, Methodology, Project administration, Visualization, Writing – original draft. FG: Formal analysis, Investigation, Methodology, Supervision, Validation, Visualization, Writing – review & editing. YL: Data curation, Investigation, Writing – review & editing. JH: Funding acquisition, Project administration, Resources, Supervision, Validation, Writing – review & editing.
